# Survival According to Therapy Regimen for Small Intestinal Neuroendocrine Tumors

**DOI:** 10.3390/jcm11092358

**Published:** 2022-04-22

**Authors:** Christine Koch, Cornelia Bambey, Natalie Filmann, Marc Stanke, Oliver Waidmann, Gabriele Husmann, Joerg Bojunga

**Affiliations:** 1 Department of Gastroenterology, Hepatology and Endocrinology, University Hospital, Goethe University Frankfurt, 60590 Frankfurt, Germany; cornelia.bambey@kgu.de (C.B.); oliver.waidmann@chop-studien.de (O.W.); joerg.bojunga@kgu.de (J.B.); 2 Department of Biostatistics and Mathematical Modelling, Goethe University Frankfurt, 60590 Frankfurt, Germany; filmann@med.uni-frankfurt.de; 3 Freelance, Sanddornweg 3, 69469 Weinheim, Germany; marcstanke@gmx.de; 4 Centrum für Hämatologie und Onkologie Bethanien, Im Prüfling 17-19, 60389 Frankfurt, Germany; 5 Tumor Documentation, University Cancer Center, University Hospital, Goethe University Frankfurt, 60590 Frankfurt, Germany; gabriele.husmann@kgu.de

**Keywords:** neuroendocrine, diagnosis, delay, metastases

## Abstract

Introduction: Scarce data exist for therapy regimens other than somatostatin analogues (SSA) and peptide receptor radiotherapy (PRRT) for siNET. We analyzed real world data for differences in survival according to therapy. Patients and methods: Analysis of 145 patients, diagnosed between 1993 and 2018 at a single institution, divided in treatment groups. Group (gr.) 0: no treatment (*n* = 10), gr 1: TACE and/or PRRT (*n* = 26), gr. 2: SSA (*n* = 32), gr. 3: SSA/PRRT (*n* = 8), gr. 4: chemotherapy (*n* = 8), gr. 5: not metastasized (at diagnosis), surgery only (*n* = 53), gr. 6 = metastasized (at diagnosis), surgery only (*n* = 10). Results: 45.5% female, median age 60 years (range, 27–84). A total of 125/145 patients with a resection of the primary tumor. For all patients, 1-year OS (%) was 93.8 (95%-CI: 90–98), 3-year OS = 84.3 (CI: 78–90) and 5-year OS = 77.5 (CI: 70–85). For analysis of survival according to therapy, only stage IV patients (baseline) that received treatment were included. Compared with reference gr. 2 (SSA only), HR for OS was 1.49 (*p* = 0.47) for gr. 1, 0.72 (*p* = 0.69) for gr. 3, 2.34 (*p* = 0.19) for gr. 4. The 5 y OS rate of patients whose primary tumor was resected (*n* = 125) was 73.1%, and without PTR was 33.3% (HR: 4.31; *p* = 0.003). Individual patients are represented in swimmer plots. Conclusions: For stage IV patients in this analysis (limited by low patient numbers in co. 3/4), multimodal treatment did not significantly improve survival over SSA treatment alone. A resection of primary tumor significantly improves survival.

## 1. Introduction

Neuroendocrine tumors of the small intestine (siNET) are a subgroup of gastroenteropancreatic neuroendocrine tumors (GEP NET), a rare entity with an incidence of 2.5/100,000 per year in Europe [1,2]. Among the GEP NET, siNET comprise about one third of cases, rendering them the largest subgroup together with the pancreatic NET (pNET) [1]. 

siNET are separated in different groups according to their grading, which is mainly deducted from a positive staining for the proliferation marker Ki67 and, to a lesser extent, the mitotic count [3]. Well-differentiated tumors with a Ki67 index of <3% are classified as G1 and, with a Ki67 of 3–20%, as G2. Poorly differentiated neuroendocrine neoplasms (NEN) with a Ki67 index above 20% are, based on morphological features, divided into NET G3 and small- or large-cell neuroendocrine carcinomas (NEC) with implications for treatment and prognosis [3]. 

In general, treatment options for small intestinal NEN depend on their grading, presence and localization of metastases and somatostatin receptor expression as assessed by functional imaging, such as somatostatin receptor scintigraphy or somatostatin receptor-directed positron emission tomography–computed tomography (PET-CT) with specific tracers such as ^68^Ga-DOTATOC or ^68^Ga-DOTATATE [4,5,6]. Some treatment modalities for NETs have been investigated in large phase II and III clinical trials; these include surgery for localized and metastatic disease, systemic treatment with somatostatin receptor analogues, or tyrosine kinase inhibitors in metastatic patients, as well as peptide receptor radionuclide therapy (PRRT) in patients with a positive somatostatin receptor expression on the tumor cells [7,8,9,10,11,12,13]. In individualized treatment protocols for selected patients or for high-grade tumors (G3), systemic chemotherapy is also an option [14,15,16,17]. Locoregional treatment such as transarterial chemoembolization (TACE) can be used in selected patients with predominantly liver metastases or treatment refractory carcinoid syndrome [18,19]. 

However, since patients with siNET often have a favorable prognosis with 5- and 10-year OS of 67 and 37%, respectively, as a recent meta-analysis reported [20], and undergo several lines of different treatments, the significance of the aforementioned results from clinical trials for real-world situations is sometime difficult to translate, especially in patients beyond classical first- or second-line treatments. In addition, some patients develop metastases with growth patterns that differ from the initial histological result with the need for individualized treatment plans.

The aim of our study was, thus, to assess survival according to treatment in patients with siNET, with an emphasis on metastasized patients and with regard to imaging and laboratory modalities in a real-life setting in a tertiary referral center. 

## 2. Patients and Methods

### 2.1. Study Design

The present retrospective, single center study was performed to investigate the survival according to therapy in patients with siNET. The study was approved by the institutional review board (internal reference number 319/16) of the University Hospital Frankfurt. Informed consent to participate in the tumor documentation registry was obtained from all living patients. Inclusion criteria of the study were diagnosis with siNET and age ≥ 18 years. 

### 2.2. Patient Data

The study database was based on the local electronic hospital charts and was transferred to the local tumor documentation system (Giessener Tumordokumentationssystem, GTDS). A specific NET data set was designed and used for documentation of all patients. The data set included epidemiological and clinical data from 1993 and is explained in detail in Supplementary Table S1. The database has been used for additional analyses without thematically overlapping [21] Data closure and end of follow-up was 15 March 2019).

### 2.3. Swimmer Plot

The data transformation, data cleansing and plotting algorithm was implemented as interactive Python Notebook (ipynb) in JupyterLab 3.0, using the python 3.7 language with matplotlib 3.2.2, pandas 1.2.0 and numpy 1.19.5. Data operations and the plotting algorithm can be found in the Swimmer_plot.ipnb in the Supplementary Materials.

### 2.4. Statistical Analyses

Statistical analyses were performed according to international standards and have been described by us and others previously. Analysis was carried out using International Business Machines Corporation (IBM) Statistical Package for the Social Sciences (SPSS) for Windows (version 22.0; IBM, Chicago, IL, USA), BiAS (version 11, Frankfurt, Germany), and R (version 3.5.1, Vienna, Austria). Categorical variables were described in frequencies and percentages. Continuous variables were represented as a median and range. Continuous variables were compared using the Wilcoxon–Mann–Whitney-U test. All tests were two-sided and *p*-values ≤ 0.05 were considered statistically significant. 

## 3. Results

### 3.1. Demographics

**Figure 1:**
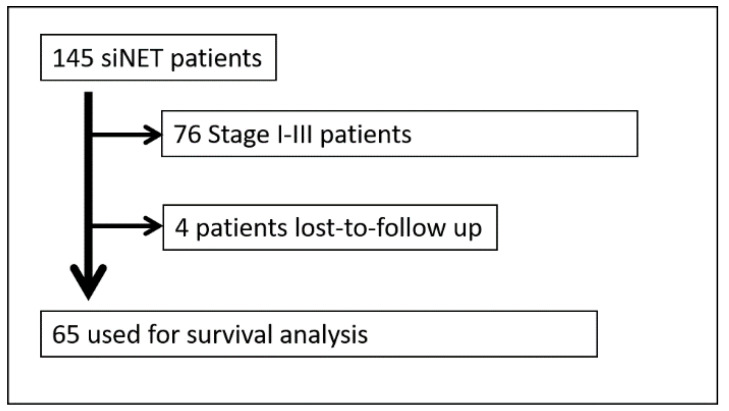
CONSORT diagram.

A total of 145 patients were included in the analysis (Figure 1). A total of 45.5% of all patients were female; the median age was 60 years (range, 27–84). The primary tumor was located in 17.9% in the duodenum; in 6.9% in the jejunum; in 63.4% in the ileum; in 0.7% in Meckel′s diverticulum; and in 11.0%, the primary location was NOS (not otherwise specified) in the small intestine. 

### 3.2. Survival According to Therapy

**Figure 2:**
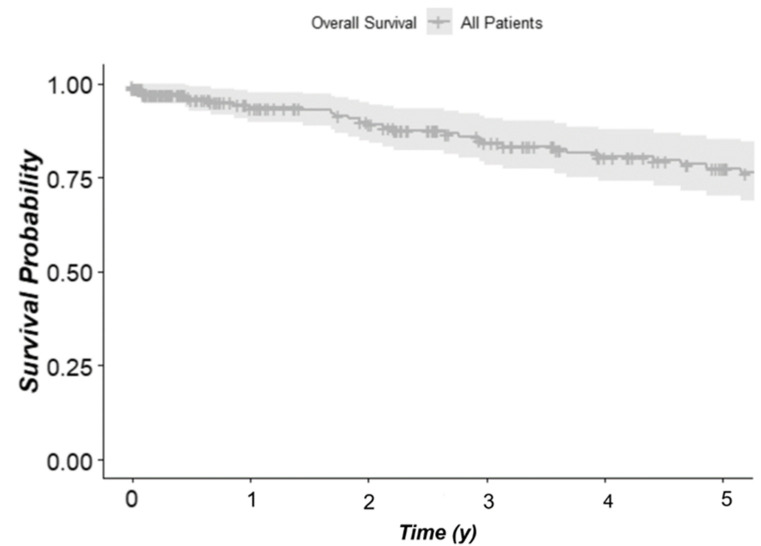
Survival probability, Kaplan–Meier analysis; median overall survival (mOS) was 17.13 years (95%-CI: 8.99–NA years).

First, we sought to analyze the survival of all patients with siNET. One-, three- and five-year overall survival probability of all patients regardless of their treatment was 93.7%, 84.3% and 77.5%, respectively. The median overall survival (mOS) was 17.13 years (95%-CI: 8.99-NA years), and the median progression-free survival (mPFS) of all patients was 6.21 years (95%-CI: 3.95–8.46 years) (Figure 2).

However, since NET patients often undergo several treatment lines over many years, which might be overlapping (e.g., somatostatin analogs and locoregional treatment), paused for a certain time or repeated after a few years, which might influence their survival, patients were divided into cohorts depending on the different regimens. Patients in group 5 were not metastasized at diagnosis, and all other patients had metastases. 

**Figure 3:**
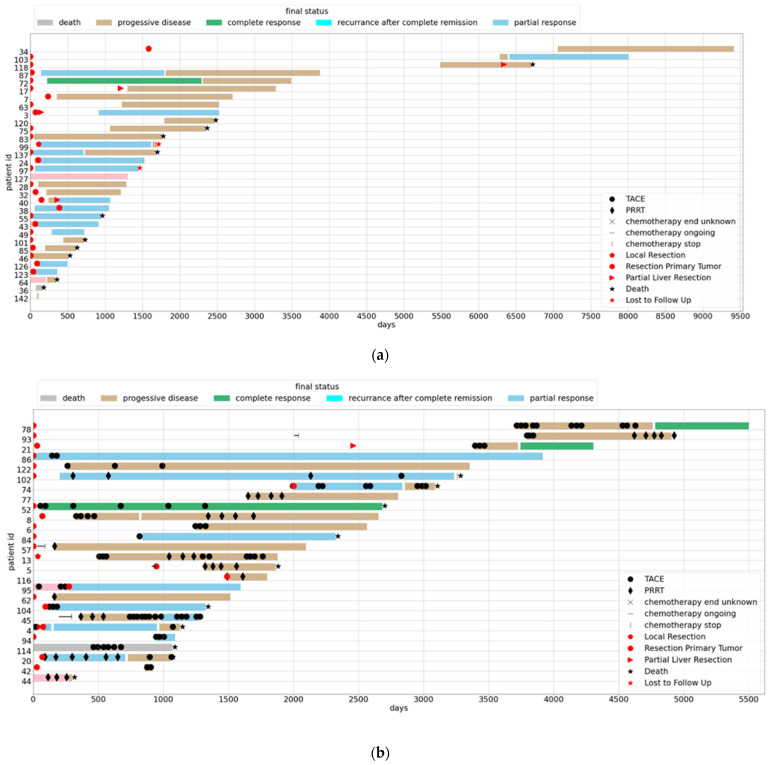

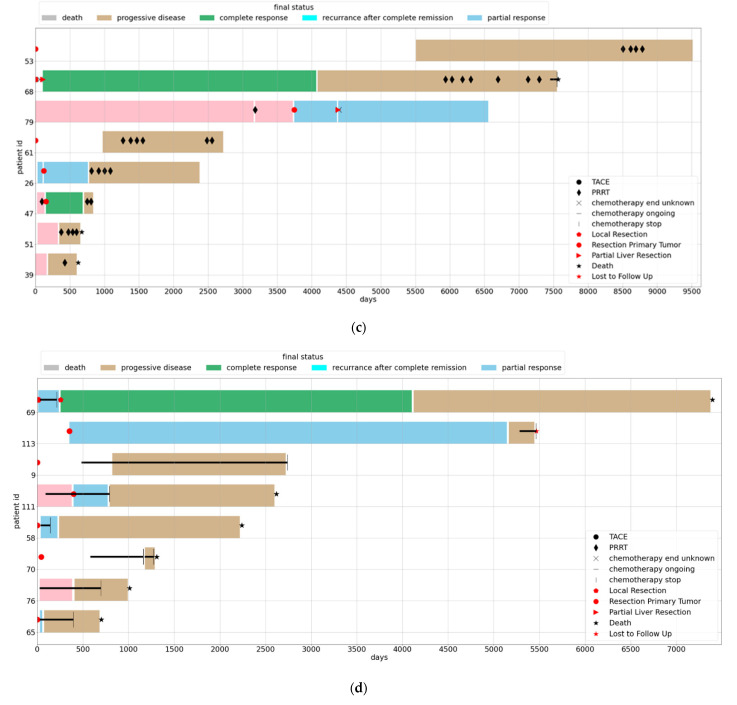

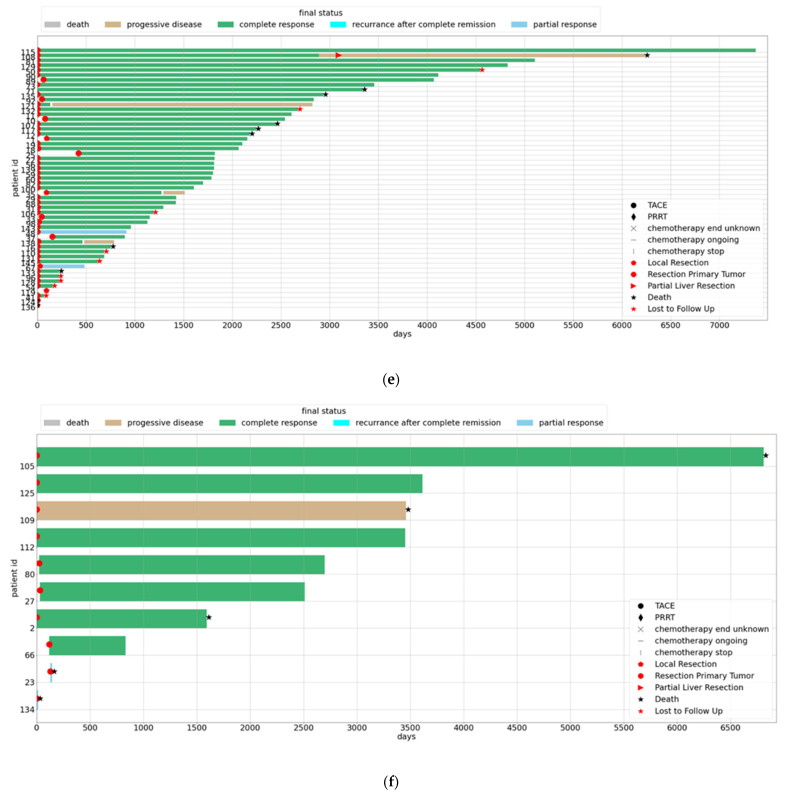
(**a**): Swimmer plot, group 1: patients (*n* = 32) treated with SSA alone; colors denote the final status (details in the figure legend), symbols denote different treatments. (**b**): Swimmer plot, group 2: patients (*n* = 26) treated with SSA parallel with TACE and/or PRRT; colors denote the final status, symbols denote different treatments. (**c**): Swimmer plot, group 3: patients (*n* = 8) treated with SSA followed by PRRT; colors denote the final status, symbols denote different treatments. (**d**): Swimmer plot, group 4: patients (*n* = 8) treated with chemotherapy; colors denote the final status, symbols denote different treatments. (**e**): Swimmer plot, group 5: patients (*n* = 53) not metastasized at diagnosis, local resection; colors denote the final status, symbols denote different treatments. (**f**): Swimmer plot, group 6: patients (*n* = 10) metastasized at diagnosis, surgery only; colors denote the final status, symbols denote different treatments.

Group 0: no treatment (*n* = 8), group 1: SSA parallel with TACE and/or PRRT (*n* = 26); group 2: SSA (*n* = 32); group 3: SSA followed by PRRT (*n* = 8); group 4: chemotherapy (*n* = 8); group 5: not metastasized (at diagnosis), surgery only (*n* = 53); group 6 = metastasized (at diagnosis), surgery only (*n* = 10). Each patient’s treatment sequence is visualized in Figure 3a–f by a “swimmer plot”. Group 0 was excluded from further analyses due to insufficient follow up data (e.g., lost to follow-up, only imaging in our clinic without further contact, only second opinion). The median time of follow up for metastasized patients (*n* = 65) was 2584 days (IQR: 1324; 3634).

**Figure 4:**
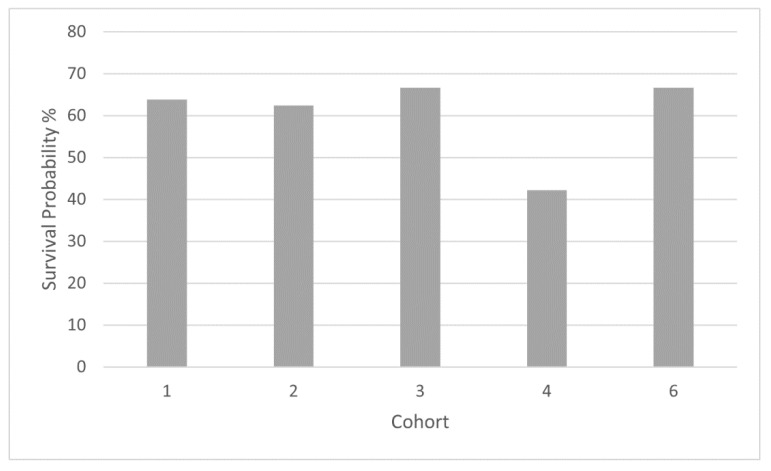
Five-year overall survival probability, groups 1–4 and 6 (metastasized patients) as percentages.

**Figure 5:**
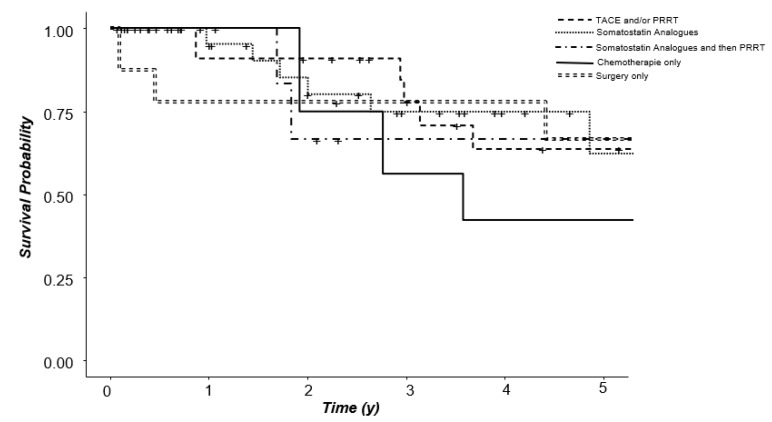
Survival probability, Kaplan–Meier analysis; reference group 2 (SSA only).

When comparing survival in the different cohorts that included stage IV patients, five-year overall survival probability was 63.8% in group 1, 62.4% in group 2, 66.7% in group 3, 42.2% in group 4 and 66.7% in group 6 (Figure 4). Survival probability shown as Kaplan–Meier curves of groups 1, 2, 3, 4 and 6 are shown in Figure 5. Hazard ratios for survival with reference group 2 (SSA only) were 1.49 (*p* = 0.467) for group 1, 0.715 (*p* = 0.693) for group 3, 2.34 (*p* = 0.191) for group 4, and 1.07 (*p* = 0.920) for group 6. 

### 3.3. Survival According to Ki67 Index

**Figure 6:**
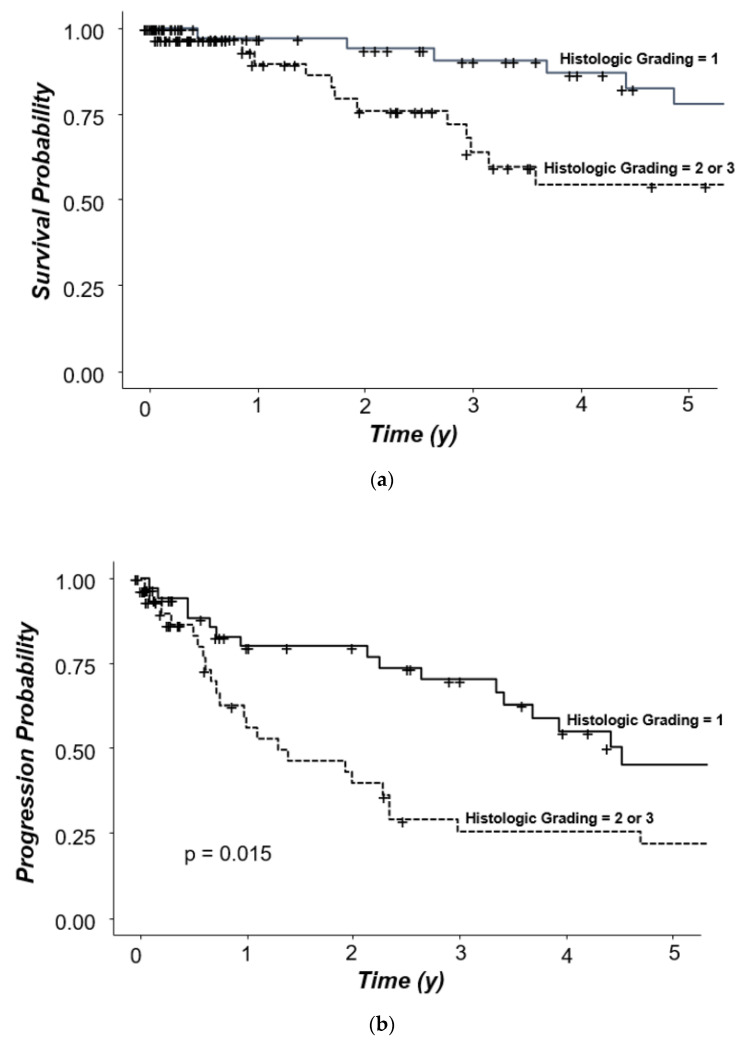
(**a**): Survival probability according to histological grading, all patients. (**b**): Progression probability according to histological grading, all patients.

The choice of treatment for siNET patients depends, among others, on the Ki67 index. Therefore, we also analyzed survival according to the respective grading (the WHO classification of 2010). An exact Ki67 staining result was available for 107/145 (73.8%) patients; grading (partly without an exact Ki67 staining result) was available for 129/145 (89%) patients. Five-year overall survival of patients with a G1 tumor (*n* = 84) was 77.9% and, thus, longer if compared to 54.2% in patients with G2 (*n* = 43) or G3 (*n* = 2) tumors (HR: 2.23; *p* = 0.061; Figure 6a). Progression-free survival rate in patients with G1 tumors was 45.4% significantly longer as compared to 21.8% in patients with a G2 or G3 tumor (HR: 2.06; *p* = 0.015; Figure 6b). 

### 3.4. Survival According to the Resection of Primary Tumor

**Figure 7:**
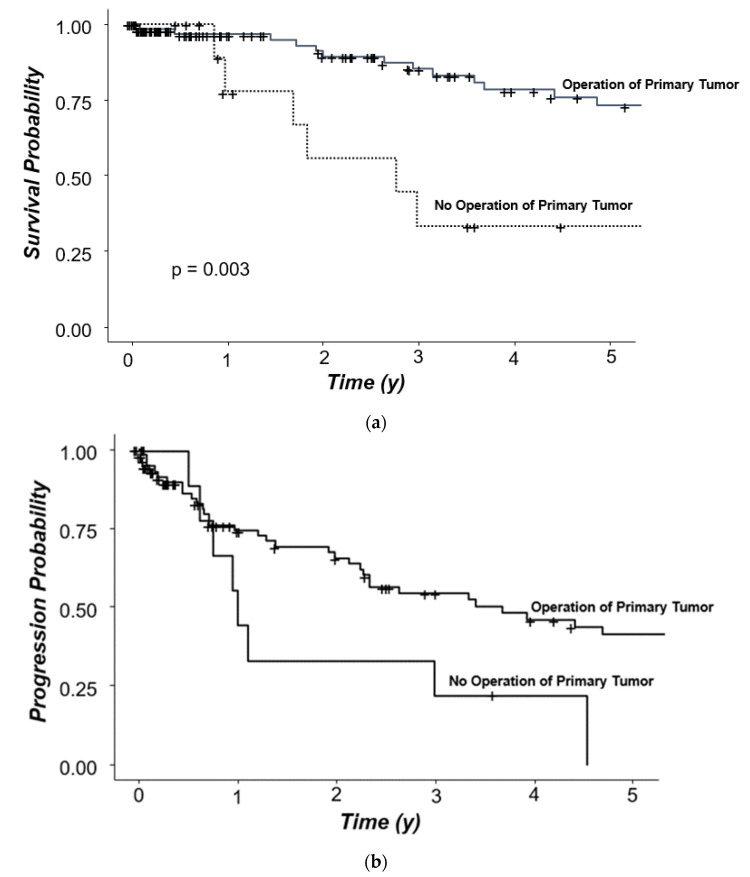
(**a**): Survival probability according to resection of the primary tumor, all patients. (**b**): Progression probability according to resection of the primary tumor, all patients.

In siNET, the resection of the primary tumor might be of benefit for the patients also in a metastasized stage. Therefore, we also analyzed survival in patients with or without resected primary tumor. Five-year overall survival of patients whose primary tumor was resected (*n* = 125) was 73.1% and, thus, significantly longer if compared to 33.3% in patients without a resection of their primary tumor (HR: 4.31; *p* = 0.003; Figure 7a). Three-year progression-free survival of patients whose primary tumor was resected was 41.3% vs. 22.2% in non-resected patients and, thus, significantly longer (Figure 7b; HR: 2.27 *p* = 0.041).

## 4. Discussion

In this large cohort of patients, we analyzed survival according to treatment in patients with siNET, a rare tumor and orphan disease. We found that, in line with the literature, patients with low-grade NET had a better survival than patients with intermediate- or high-grade tumors, and that patients benefitted from a resection of their primary tumor [22,23,24,25]. 

However, when analyzing survival according to treatment regardless of the Ki67 index, the picture is quite diverse. Patients often underwent long-term treatments with prolonged phases without any therapy. Furthermore, for most patients, there was no clear path as other malignant diseases with, e.g., first treatment A, followed by treatment B and so forth. Instead, most patients received different treatments such as locoregional (TACE) or systemic treatments in different order. Many patients underwent surgery for localized or metastatic disease at some point of time, and most received SSA treatment. Taken together, we decided to split the whole cohort in different subgroups according to the predominant therapeutic regimen and visualized each patient′s pathway by a line in a classical swimmer plot. One limitation of the study clearly is that, due to the retrospective design and the large time span, a clear division of all patients was not possible. Therefore, we chose based on the predominant treatment the patient received for the majority of time. Similar analyses have been published from the Swiss NET registry but without survival analyses [26].

Most metastasized patients in our cohort were treated with a combination of SSA and locoregional treatments, either PRRT or TACE (for liver-dominant tumors) or both. SSA followed by PRRT upon progression was administered to eight patients only. This approach was recently prospectively investigated in the NETTER-1 trial [11], showing a clear survival benefit for patients with disease progression treated with PRRT over patients with dose escalation of the SSA (PFS: NR vs. 8.4 months; *p* > 0.001; HR 0.21). 

Interestingly, the five-year survival probability in our cohort was comparable between all groups except for the patients that received chemotherapy who showed a shorter survival time, which was also observed by Faggiano et al. in a mixed cohort of G1/2 NET patients [27]. They split up a cohort of 99 patients with ≥2 lines of treatment into four different groups according to the therapeutic sequence, and analyzed PFS of first- and second-line treatment. Interestingly, there was no significant difference in either line for each group in this retrospective multicenter analysis. Toxicity, however, was higher in patients receiving either chemotherapy or everolimus. Chemotherapy in siNET is mainly administered in patients with G2 or G2 tumors with a Ki67 > 20%. In our cohort, there were only two patients with a G3 tumor; the remaining patients were G1/2. However, due to the low number, no clear interpretation is possible here. 

The role of cytotoxic chemotherapy in siNET patients is still debatable since only small series of patients with GEP-NET that received chemotherapy are described in the literature, often mixed groups of patients with different primary tumors or combination treatments [28]. 

Shortcomings of our study are the retrospective and single-center nature, although we were able to analyze a large data set over a long period. However, due to possibly incomplete data and difficult comparability of different subgroups as outlined above, survival analyses have to be interpreted with caution. 

Taken together, we show in this retrospective trial that treatment of siNET is rarely carried out in a strictly linear manner and involves several therapeutic options. Survival, however, is comparable between the groups and depends mainly on the resection of the primary tumor and grading.

## Data Availability

The data presented in this study are available on request from the corresponding author. The data are not publicly available due to ethical and legal restrictions. Aleniated data used for the swimmer plot are stored in a public repository: https://gitlab.com/mstanke/swimmers_plot (accessed on 15 April 2022).

## Supplementary Materials

The following supporting information can be downloaded at: https://www.mdpi.com/article/10.3390/jcm11092358/s1, Table S1: Basic data set according to ADT/GEKiD with UCT-specific additions.
